# The vaginal isolate *Lactobacillus paracasei* LPC-S01 (DSM 26760) is suitable for oral administration

**DOI:** 10.3389/fmicb.2015.00952

**Published:** 2015-09-15

**Authors:** Silvia Balzaretti, Valentina Taverniti, Greta Rondini, Giorgio Marcolegio, Mario Minuzzo, Maria C. Remagni, Walter Fiore, Stefania Arioli, Simone Guglielmetti

**Affiliations:** ^1^Division of Food Microbiology and Bioprocessing, Department of Food Environmental and Nutritional Sciences, Università degli Studi di MilanoMilano, Italy; ^2^Department of Biosciences, Università degli Studi di MilanoMilano, Italy; ^3^Sofar S.p.A.Trezzano Rosa, Italy

**Keywords:** lactobacilli, probiotic, Caco-2 adhesion, NF-κB, *in vivo* trial, gastrointestinal persistence

## Abstract

Bacterial vaginosis is one of the most common urogenital diseases affecting women in reproductive age. The administration of probiotics as vaginal suppository has been proposed as a strategy to cure this condition and reduce its recurrence. Nonetheless, also oral consumption of probiotics, which is a more practical route of administration, proved to be an efficient strategy. In this perspective, we studied *Lactobacillus paracasei* LPC-S01 (DSM 26760), a human vaginal isolate included in commercial probiotic preparations for topical use, in order to assess if this bacterium can also perform as gastrointestinal probiotic. Comparative genomics revealed the presence of several accessory genes suggesting that LPC-S01 is a niche-generalist member of its species. According to a procedure conventionally used to predict the probiotic potential, we demonstrated that the probiotic properties of strain LPC-S01, with respect to those of the well-known probiotic references *L. paracasei* Shirota and DG, are equal for the bile tolerance and the reduction of NF-κB activation in Caco-2 cells, or superior for the tolerance to gastric juice and the adhesion to Caco-2 epithelial cells. We then demonstrated that LPC-S01 is susceptible to antibiotics indicated by EFSA and does not produce biogenic amines. Finally, a double-blind cross-over pilot intervention trial on healthy human volunteers showed that, after a 7-days oral consumption of capsules containing about 24 billion live cells, the fecal cell concentrations of strains LPC-S01 and DG (evaluated by qPCR) were not dissimilar. Specifically, both probiotics' cell concentrations were above the detection limit for an average of 5 days from the end of the treatment, corresponding to a mean number of evacuations of 7 ± 2. Taken together, these data demonstrate that the vaginal isolate *L. paracasei* LPC-S01 possesses safety and functional properties that may support its use as probiotic to be administered *per os* for potential intestinal as well as vaginal applications.

## Introduction

The genus *Lactobacillus* is a taxonomically broad and heterogeneous group of Gram positive bacteria, which has important industrial applications as fermented food starter and probiotic adjuncts. Particularly, lactobacilli are the bacteria most frequently employed as probiotics, i.e., “live microorganisms which when administered in adequate amounts confer a health benefit on the host” (FAO/WHO, 2002). Inside the *Lactobacillus* genus, the *L. casei* group of species (i.e., *L. casei, L. paracasei*, and *L. rhamnosus*) includes some of the most conventional and well-characterized probiotic strains, such as *L. paracasei* Shirota (considered the first probiotic ever; Nanno et al., 2011), and *L. rhamnosus* GG (Szajewska and Chmielewska, 2013).

Strains of the *L. paracasei* species have been isolated from several diverse ecological niches such as raw milk, plants, fermented artisanal products (fermented milk, cheese, sourdough bread starter, and fermented vegetables), and the intestinal tracts and reproductive systems of humans and animals (Cai et al., 2007). The remarkable ecological adaptability of *L. paracasei* to diverse habitats can be plausibly explained by very frequent accumulation of indels and genome rearrangements, which originated a high level of genotypic and phenotypic diversity in the species (Cai et al., 2007; Smokvina et al., 2013). Given that most effects of probiotics are strain-specific (Azaïs-Braesco et al., 2010), the intrinsic high heterogeneity existing among *L. paracasei* strains makes this species an optimal source for the selection of novel candidate probiotic strains possessing unique technological and health-promoting traits.

So far, lactobacilli have been predominantly investigated and explicitly proposed as probiotics for their potential beneficial roles on the gastrointestinal tract and its microbiota (Guglielmetti et al., 2011; Ferrario et al., 2014). Nevertheless, the interest for the application of lactobacilli beyond the gut is constantly increasing, leading to the development of new categories of probiotic products that target oral cavity (Guglielmetti et al., 2010a,b; Taverniti et al., 2012; Wescombe et al., 2012), skin (Krutmann, 2009), stomach (Johnson-Henry et al., 2004), urinary tract (Borchert et al., 2008), and vaginal mucosa (Borges et al., 2014).

Interestingly, it was demonstrated that orally administered lactobacilli can be re-isolated from the vagina, presumably as a consequence of the migration from the rectum via perineum (Vásquez et al., 2005). Consistently, bacteria colonizing the vaginal mucosa (both commensals and vaginosis-associated microbes) have been isolated from the rectum and the mouth, suggesting that gut and oral cavity act as extravaginal reservoirs of vaginal microbiota bacteria (van de Wijgert et al., 2014). Therefore, it appears plausible that the oral administration of probiotic bacteria may potentially influence the vaginal microbiota through two possible mechanisms: (i) modification of the intestinal microbiota (e.g., by reducing potentially harmful bacteria and increasing endogenous lactobacilli); (ii) direct migration to the vaginal mucosa via the gastrointestinal route. Furthermore, the possibility to benefit vaginal health by administering vaginal probiotics *per os* has the additional advantage of favoring the accomplishment of long-term treatments. However, in order to make this strategy effective, selected bacteria must possess properties supporting their survival and activities both in the gastro-intestinal tract and vaginal mucosa. In this perspective, we assessed if *L. paracasei* LPC-S01 (DSM 26760) (a strain originally isolated from the vaginal mucosa of a healthy woman and included in probiotic preparations for vaginal use marketed in Europe) can also perform as a conventional gastrointestinal probiotic. To this aim, we generated a draft genome of strain LPC-S01 and performed a comparative genomic analysis against other *L. paracasei* reference strains. In addition, we included LPC-S01 in several *in vitro* tests conventionally employed to establish the intestinal probiotic potential of microbial strains. Finally, we carried out a double-blind cross-over pilot intervention trial in comparison with the well-known intestinal probiotic strain *L. paracasei* DG (Ferrario et al., 2014) in order to assess the ability of LPC-S01 to transiently persist in the gastrointestinal tract of healthy adults.

## Materials and methods

### Bacterial strains and culture conditions

Unless differently specified, lactobacilli were grown at 37°C in De Man-Rogosa-Sharpe (MRS) broth (Difco Laboratories Inc., Detroit, MI) for 24 h. *L. paracasei* LPC-S01 and DG were isolated from the commercial products PreGyn® and Enterolactis® Plus (Sofar S.p.A.), respectively; *L. paracasei* strain Shirota was isolated from Yakult fermented milk; strain FMBr3 was isolated from Raschera artisanal Italian cheese.

### Genome sequencing, sequence annotation, and comparative analysis

The draft genome sequences of *L. paracasei* LPC-S01, DG, and FMBr3 were obtained through Ion Torrent PGM (Life Technologies, Germany) according to a previously described protocol (Milani et al., 2013) by GenProbio Ltd. The raw sequence data were assembled using MIRA v.3.9 (http://www.chevreux.org/projects_mira.html), applying default parameters recommended for Ion Torrent data processing. Initial automated annotation of the *L. paracasei* genomes was performed using RAST, combined with BLASTX. Results of the gene-finder program were combined manually with data from BLASTP analysis against a non-redundant protein database provided by the National Center for Biotechnology Information (NCBI). LPC-S01 draft genome sequence was compared with other *L. paracasei* genome sequences by means of BLAST Ring Image Generator (BRIG; Alikhan et al., 2011). The DNA sequences presented in this study have been deposited in the EMBL database under the accession numbers LN846896 (putative phospho-β-galactosidase operon), LN846897 (putative taurine ABC-type transport and metabolization operon), LN846898 (putative ABC-type Fe3+ transport system), LN846899 (putative nucleotide transport and metabolism operon), LN846900 (putative EPS2 region), and LN846901 (multi-transport region for the uptake of sugars and other small molecules).

### Bacterial resistance to simulated gastric juices and bile

One hundred microliters of *L. paracasei* culture containing 10^8^–10^9^ CFU/ml were transferred to 6 ml of simulated gastric juice at pH 2 (1 g/l DIFCO peptone, 100 mM KCl, 500 U/ml pepsin) or simulated gastric juice at pH 3 (125 mM NaCl, 45 mM NaHCO_3_, 7 mM KCl, 500 U/ml pepsin). Cells in 0.1 M phosphate buffer pH 7 were used as control. After 90 min of incubation at 37°C, viability was monitored by plating on MRS agar. Afterwards, cell suspension was neutralized by adding 1 M phosphate buffer pH 8 and centrifuged at 10,000 × g for 12 min. Bacterial cell pellet was then suspended in 5 ml bile solution (1 g/l peptone, 0.3% Difco Oxgall in 0.1 M phosphate buffer at pH 6.5). Cells in 0.1 M phosphate buffer pH 6.5 in the presence of 1 g/l peptone were used as control. The number of viable cells was determined after 3 h of incubation at 37°C by plating on MRS agar. Plates were incubated under anaerobic conditions and colonies were counted after 48 h. The ability to grow in presence of bile was also investigated by inoculating bacteria in MRS broth supplemented with increasing concentrations of Oxgall (from 0.0625 to 9.6%). Growth was assessed by measuring the optical density at 600 nm.

### Bacterial adhesion to Caco-2 cell line

The adhesion of *L. paracasei* strains to Caco-2 (ATCC HTB-37) cell layer was assessed as previously described (Guglielmetti et al., 2008). In brief, Caco-2 cells were grown in Dulbecco's Modified Eagle's Medium (MEM) supplemented with 10% (v/v) heat-inactivated fetal calf serum, 100 U/ml penicillin, 100 mg/ml streptomycin, 0.1 mM non-essential amino acids, 2 mM L-glutamine and incubated at 37°C in an atmosphere of 95% air and 5% carbon dioxide. For adhesion experiments, fully differentiated Caco-2 were used (i.e., 15 days after confluence). Approximately 2 × 10^8^ cells for each bacterial strain (determined microscopically with Neubauer Improved counting chamber; Marienfeld GmbH, Lauda-Königshofen, Germany) were incubated with a monolayer of Caco-2 cells for 1h at 37°C. Monolayers were washed three times with phosphate-buffered saline pH 7.3 (PBS) to release unbound bacteria and incubated with 3 ml of methanol for 8 min at room temperature to fix cells. Afterwards, cells were stained with 3 mL of Giemsa stain solution (1:20) (Carlo Erba, Milano, Italy) and left 30 min at room temperature in the dark. Finally, monolayers were washed three times with PBS, dried in an incubator for 1 h, and examined microscopically (magnification, 400 ×) under oil immersion. All experiments were performed in duplicate.

### Inhibition of pathogens

We investigated the ability of *L. paracasei* strains to secret inhibitor molecules against pathogens by disc diffusion assay. *Lactobacillus plantarum* strain WHE 92 was used as a positive control for bacteriocin production (Ennahar et al., 1996). We used seven indicator microorganisms: *Salmonella enterica* MIMms, *Listeria monocytogenes* FMB4b, *Escherichia coli* VE7108, *Streptococcus pyogenes* emm TYPE 77, *Pseudomonas aeruginosa* FMBpa18, *Staphylococcus aureus* FMBDR, and *Candida albicans* ATCC MYA2876. Indicator bacteria were cultivated in Brain Heart Infusion (BHI) agar medium (Oxoid S.p.A., Milan, Italy), supplemented with 0.3% yeast extract and 1% glucose (ygBHI), whereas *C. albicans* was cultivated on Sabouraud dextrose agar (Oxoid S.p.A., Basingstoke, UK). Three different fractions for each *Lactobacillus* strain (tester bacteria) were used: (i) broth culture at stationary growth phase, (ii) cell-free broth (obtained by centrifugation at 10,000 × g for 10 min and sterilization with a 0.22 μm syringe filter), and (iii) cell-free broth neutralized with NaOH to pH 7.4. Sterile MRS was used as negative control. The assay was carried out as follows: 0.1 ml from an over-night culture of the indicator strains were plated on ygBHI (or Sabouraud) agar in order to obtain a confluent growth after incubation. Sterile paper discs (Whatman™ Grade AA 9 mm disc, Maidstone, UK) were dipped into three different preparations from tester bacteria and subsequently positioned on the Petri dishes inoculated with the indicator strain. Plates were kept at 4°C for 2 h and then incubated at 37°C. The presence of an inhibition halo was checked after 24 or 48 h.

### Fermentation profile

Carbon source fermentation was determined in a 96-well microtiter plate in a final volume of 200 μl with a basal CHL medium at pH 6.3 (Bio-Merieux, Montelieu-Vercieu, France) containing bromocresol purple as pH indicator, and the desired filter-sterilized carbohydrate at a final concentration of 0.5% (w/v). The 42 different substrates tested (Supplementary Table 1) were from Sigma-Aldrich (St. Louis, MO, USA), with the exception of fructooligosaccharides (FOS) which were from Actilight® (Giulio Gross S.p.A., Trezzano sul Naviglio, Italy). Cells from an over-night culture of *L. paracasei* were collected by centrifugation, washed with PBS, and used to inoculate the liquid medium in microtiter wells (1/100 inoculation). Plates were examined for color change (from purple to yellow) after 24 and 48 h incubation at 37°C.

### NF-κB activation assay

The activation nuclear factor κB (NF-κB) was studied by means of a recombinant Caco-2 cell line stably transfected with vector pNiFty2-Luc (InvivoGen, Labogen, Rho, Italy) as in Taverniti et al. (2013). In brief, recombinant Caco-2 monolayers (approximately 3 × 10^5^ cells/well), cultivated in the presence of 50 μg/ml zeocin, were washed with 0.1 M Tris-HCl buffer (pH 8.0) and then incubated with 3.5 × 10^8^ cells of *L. paracasei* suspended in fresh DMEM containing 100 mM HEPES (pH 7.4), resulting in a MOI of approximately 1000. In a different set of experiments, Caco-2 monolayers were incubated with 0.1 ml of cell-free broth obtained by centrifugation and filter-sterilization from a stationary-phase culture of *L. paracasei*. Stimulation was conducted by adding 2 ng/ml of IL-1β. After incubation at 37°C for 4 h, the samples were treated and the bioluminescence was measured as described by Stuknyte et al. (2011). Two independent experiments were conducted in triplicate for each condition.

### Susceptibility to antibiotics and biogenic amines production

The minimal inhibitory concentrations (MICs) of *L. paracasei* have been determined using a micro-dilution method in LSM broth (ISO-Sensitest broth, Oxoid supplemented with 10% v/v MRS Difco) as described in the ISO10932 IDF 223 document and recommended by EFSA (2012). The data are reported as average of two independent assays. The ability to produce cadaverine, tyramine, histamine and putrescine respectively from L-lysine, tyrosine disodium salt, L-histidine monohydrochloride and L-ornithine monohydrochloride was investigated by a qualitative test, according to the method proposed by Bover-Cid and Holzapfel (1999). All amino acids were purchased from Sigma. Strains were inoculated (1%) in MRS broth containing 0.1% of each amino acid precursor and incubated at 37°C for 18 h. The qualitative test to evaluate decarboxylase activity was performed in an agar medium specially formulated (0.5% tryptone, 0.5% yeast extract, 0.5% meat extract, 0.25% NaCl, 0.05% glucose, 0.1% Tween 80, 0.02% MgSO4, 0.005% MnSO4, 0.004% FeSO4, 0.2% ammonium citrate, 0.001% thiamine, 0.2% K2PO4, 0.01% CaCO3, 0.005% pyridoxal phosphate, 1% amino acid precursor, 0.006% bromocresol purple, 2% agar; pH 5.3). 10 μL of each culture were placed on the medium containing the same amino acid precursor. The plates were incubated at 37°C for 4 days. Positive test is indicated by the color change and by aminoacid precipitation around the corresponding spot (for tyramine only).

### Human intervention trial

The study protocol was approved by the Research Ethics Committee of the Università degli Studi di Milano (opinion no. 37/12, December 2012). Written informed consent was obtained from all subjects before recruitment. The intervention study consisted of a randomized, double blind, cross-over pilot trial with two parallel groups (Supplementary Figure 1). Each volunteer was asked to participate to 6 visits: before run-in period (visit V0); before the first treatment (V1); after the first treatment (V2); before the second treatment (V3); after the second treatment (V4), and at the end of the trial (V5) (Supplementary Figure 1). After a 4-week pre-recruitment phase, volunteers have been randomized to receive 1 capsule daily of *L. paracasei* LPC-S01 (product A, 5 subjects) or *L. paracasei* DG (product B, 6 subjects) every day for 1 week, in addition to their habitual diet. After a 2 week wash-out period, the volunteers received a daily capsule of the other product for 1 week. Volunteers received directions to keep the products at room temperature and to avoid exposure to heat sources. Volunteers received also oral and written instructions to consume the capsule during the morning, while drinking natural water, at least 15 min before breakfast; alternatively, volunteers were allowed to consume capsule in the evening at least 3 h after the last meal of the day. The two probiotic preparations (provided by Sofar S.p.A., Trezzano Rosa, Italy) consisted of a gelatin capsule containing about 24 billion viable cells of the bacterial strain *L. paracasei* DG (CNCM I-1572) and LPC-S01 (DSM 26760), respectively; silicon dioxide and magnesium stearate were also added inside capsules as antiagglomerants. Capsules were delivered to participants in metal boxes sealed with a plastic cap containing desiccant salts. From the last day of consumption of the probiotic capsules, for the following 7 days, the volunteers provided one stool sample per day. Furthermore, participants provided a fecal sample at visits V1, V3, and V5 (Supplementary Figure 1). The sample was collected in a sterile plastic pot no more than 24 h before visit. Volunteers were asked to preserve the fecal sample at room temperature until delivery to the laboratory, according to the recommendations on storage conditions of intestinal microbiota matter in metagenomic analysis provided by Cardona et al. (2012). During the trial period, the participants compiled a weekly diary (including a Bristol stool chart) of their bowel habits.

### Fecal DNA extraction and qPCR

Stools were stored at −80°C until DNA extraction, performed by means of QIAamp DNA Stool Mini Kit (Qiagen, Valencia, CA). Before the extraction, the sample was homogenized in a Bead Beater Precellys 24 (Bertin Technologies, Montigny le Bretonneux, France) after addition of 0.45 g 0.5 mm glass beads. Subsequently, the extraction proceeded consistently with manufacturer's recommendations. Real-Time quantitative (qPCR) protocols were adopted for the quantification of *L. paracasei* DG in fecal metagenomic DNA (targeting the glycosyltransferase gene *welF*, with primers rtWELFf, 5′-TACTAAAGAAATTAGCTTTTGT-3′ and rtWELFr, 5′-AGTAATGTCTGCATCCTCCA-3′; Ferrario et al., 2014) and LPC-S01(targeting an hypothetical protein coding sequence with primers qS01a-F, 5′-TGGAAGAGACCCTGCGAA-3′ and qS01a-R, 5′-GAGGTTGATTCACAAACCGTGC-3′; this study). These two genes were selected because they resulted unique to the respective strain as revealed by a search in the GenBank nucleotide database. A gradient PCR was initially performed to standardize the qPCR conditions. qPCR amplifications were carried out in a final volume of 15 μl containing 7.5 μl of EvaGreen® Supermix and 0.5 μM of each primer. We used 100 ng of fecal DNA template in each reaction. Samples were amplified with the following programs: for rtWELF primers, initial hold at 95°C for 3 min, and 44 cycles at 95°C for 30 s, 58°C for 30 s and 72°C for 30 s; for qS01a primers, initial hold at 95°C for 3 min, and 40 cycles at 95°C for 30 s, 62°C for 30 s and 72°C for 30 s. Melting curves were analyzed to confirm the specificity of the amplification products. To generate the standard curves with spiked feces for DG and LPC-S01 detection, a 200 mg matrix of fecal sample was added with 1 × 10^9^ bacterial cells and DNA was extracted as reported above. Six-fold dilution series of isolated DNA were prepared and used in qPCR reactions. The equation of the derived standard curves was used to calculate the correlation between Ct values and the concentration of bacterial cells per g of feces.

## Results

### Comparative genomics revealed the indels of several chromosomal regions in *L. paracasei* LPC-S01

**Figure 1:**
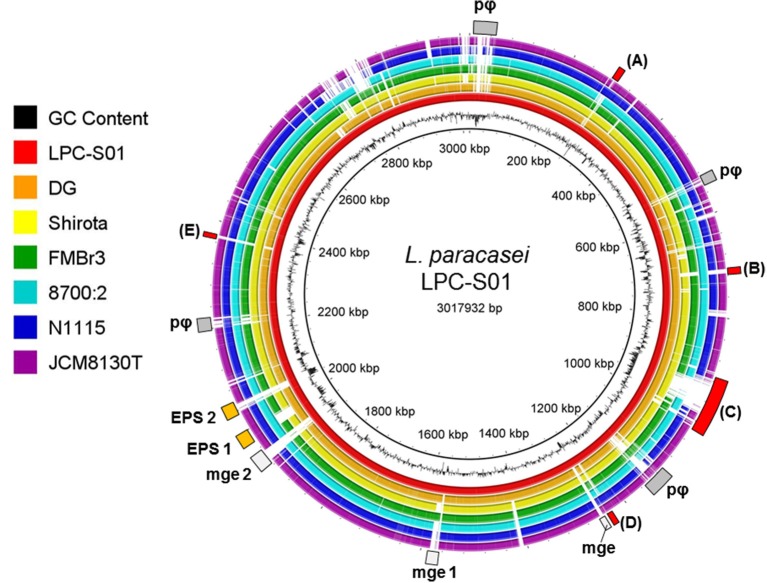
**Comparative genomic analysis of ***Lactobacillus paracasei*** LPC-S01 (reference genome) with the genome sequences of other six ***L. paracasei*** strains**. Gray rectangles indicate mobile genetic elements. pφ, prophage-related regions. mge, mobile genetic element; mge 1, putative integrated plasmid; mge 2, putative mobile genetic element containing a restriction-modification system. EPS, putative exopolysaccharide coding operon. Letters flanking red rectangles refers to panels of Figure 2.

**Figure 2:**
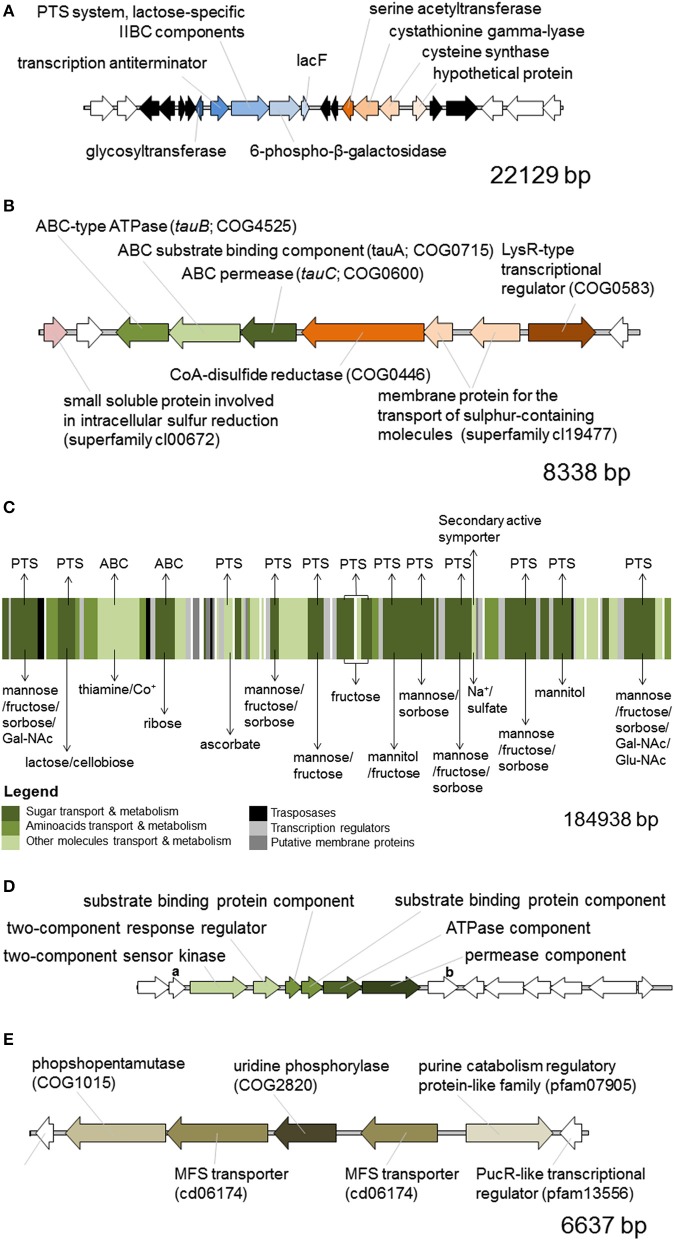
*****In silico*** predicted functional organization of the chromosomal regions of ***Lactobacillus paracasei*** LPS-S01 that discriminates LPC-S01 from the others strains included in the comparative genomic analysis depicted in Figure 1**. Letters in the panels of this figure refer to the letters flanking red rectangles in Figure 1. **(A)** Putative phospho-β-galactosidase operon. In black are indicated transposase-associated putative genes. In white are indicated putative genes that are also present in the genome of the other *L. paracasei* strains considered for comparative genomic analysis. **(B)** Putative taurine ABC-type transport and metabolization operon. **(C)** Multi-transport region for the uptake of sugars and other small molecules. The membrane transport mechanism is indicated above the picture, whereas the transported molecule is indicated below. ABC, ATP binding cassette transport system, PTS, phosphotransferase transport system. Gal-NAc, N-acetyl galactosamine; Glu-NAc, N-acetyl glucosamine; Co^+^, cobalt ions. **(D)** Putative ABC-type Fe^3+^ transport system. Gene “a,” putative acetyltransferase (COG0456) coding gene; gene “b,” putative gene coding for an uncharacterized protein (DegV family, COG1307). **(E)** Putative nucleotide transport and metabolism operon. MFS, major facilitator superfamily. In white are indicated putative genes that are also present in the genome of all the other *L. paracasei* strains considered.

**Figure 3:**
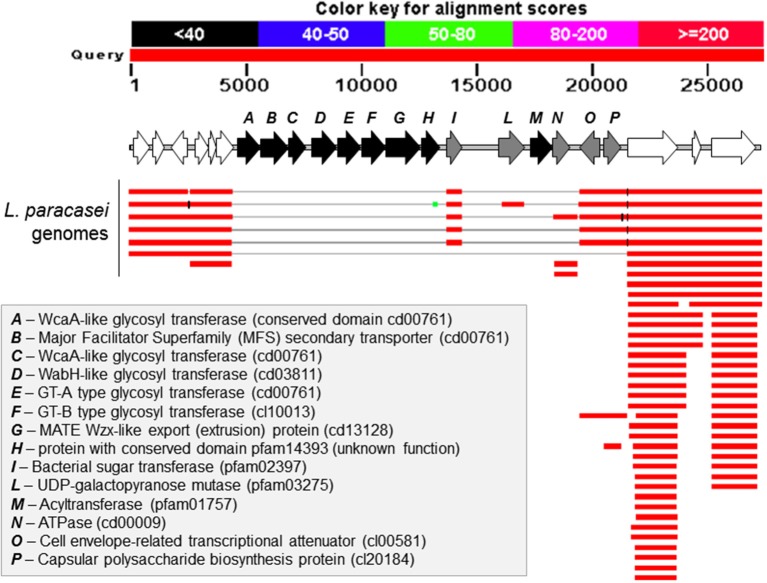
**BLASTN search results for the region EPS2 from ***Lactobacillus paracasei*** LPC-S01, which putatively codes for the enzymes involved in the synthesis of an exopolysaccharide (EPS)**. The figure has been obtained adding a picture of the putative EPS operon over the graphic representation of the BLASTN output. In white are indicated genes outside the putative EPS operon.

We generated a draft genome sequence of *L. paracasei* LPC-S01 consisting of 17 contigs, which in total contained 3.02 Mbp. Comparative genomic analysis was performed on strain LPC-S01 and the following six *L. paracasei* strains: the type strain of the species (*L. paracasei* JCM 8130^T^), the probiotic strains DG and Shirota, a strain isolated from Raschera Italian artisanal cheese (FMBr3), and two other *L. paracasei* strains whose complete genome is available in GenBank (strains 8700:2 and N1115) (Figure 1). When we used *L. paracasei* LPC-S01 as reference genome in the comparative analysis, we identified 14 genomic islands with putatively known biological functions, which have not been found in the genomes of type strain JCM 8130^*T*^ or other *L. paracasei* strains. These chromosomal islands, which discriminate LPC-S01 genome from the others, include 7 mobile genetic elements (e.g., prophages, phage remnants, and integrated plasmids; Figure 1), a transposon containing a phospho-β-galactosidase operon (region “a” in Figure 1; Figure 2A), a putative taurine transport and metabolization operon (b; Figure 2B), a large sugars transport/metabolization island (c; Figure 2C), an ABC-type Fe^3+^ transport system (d; Figure 2D), and a putative nucleotide transport and metabolism operon (e; Figure 2E). We also found in the genome of strain LPC-S01 two regions putatively involved in exopolysaccharide (EPS) synthesis. Specifically, one of these EPS regions (namely, EPS2; Figure 1) was found exclusively in the genome of *L. paracasei* LPC-S01 and is constituted of 14 putative genes, including six genes potentially coding for glycosyl transferases. Notably, BLASTN search revealed inside EPS2 a region of about 8 kb encompassing eight putative genes, which did not find any significant match in the GenBank database (genes from A to H in Figure 3). In subsequent comparative analyses, we used the genome of the other *L. paracasei* strains as reference to identify missing chromosomal islands in the genome of strain LPC-S01. Largely most of the genomic regions that are absent in LPC-S01 genome refer to mobile genetic elements, DNA restriction-modification systems, and EPS biosynthesis regions (data not shown).

### *In vitro* assays showed that *L. paracasei* LPC-S01possesses marked acid tolerance and noticeable adhesion ability

**Figure 4:**
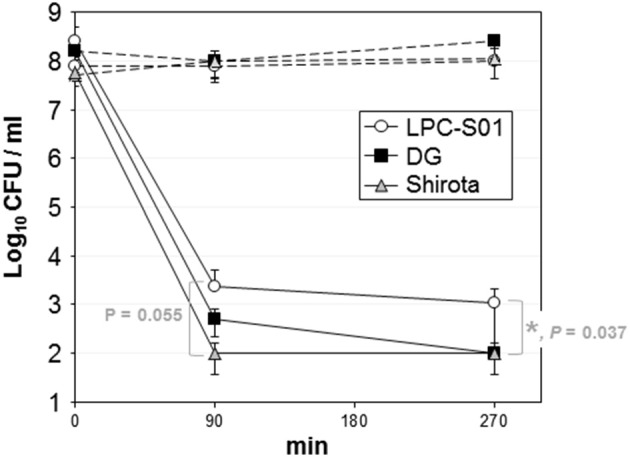
**Tolerance of ***Lactobacillus paracasei*** strains to simulated gastric juice at pH 2 (90 min incubation) and bile (180 min incubation)**. Dashed lines refer to controls (i.e., bacterial cells incubated in phosphate buffer, pH 6.5). Data are reported as the number of viable bacterial cells (CFU) plotted on a semi-logarithmic diagram. Vertical bars at each point refer to standard deviation calculated on three independent experiments conducted in duplicate. Asterisk between strain LPC-S01 and Shirota at the end of the experiment indicates statistically significant difference according to two-tailed unpaired Student's t test (^*^P < 0.05).

The probiotic potential of strain *L. paracasei* LPC-S01 was assessed through *in vitro* experiments including *L. paracasei* DG and *L. paracasei* Shirota as reference probiotic strains. The potential ability to survive the gastro-intestinal transit was assessed in simulated gastric juices at pH 3 or 2 (90 min incubation), followed by 3 h incubation in bile (Oxgall) solution. All strains showed high tolerance to pH 3; cell viability, in fact, was not significantly reduced after 4.5 h (Supplementary Figure 2). Conversely, pH 2 determined a drastic reduction of the viability of all tested strains (Figure 4). However, *L. paracasei* LPC-S01 displayed the highest tolerance, which was significantly higher than that of the reference strain *L. paracasei* Shirota (Figure 4).

Growing concentrations of Oxgall were added to the MRS broth to assess the ability to grow in presence of bile. For all tested strains, we observed a dose-dependent inhibition of the bacterial growth, which was completely arrested at Oxgall concentrations higher than 1%. In specific, Shirota displayed the best tolerance to bile, even though not statistically significant (Supplementary Figure 3).

**Figure 5:**
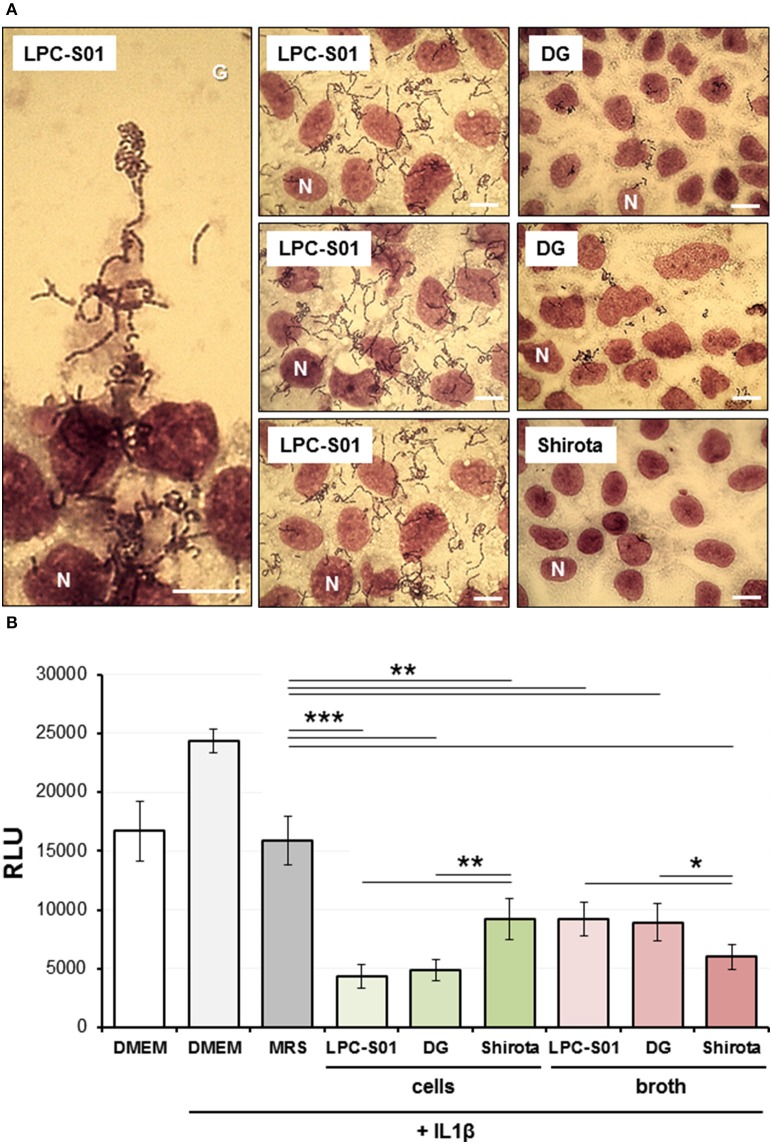
**Experiments on human epithelial colorectal Caco-2 cell layer. (A)** Adhesion of *Lactobacillus paracasei* strains to the Caco-2 epithelial cell layer as observed with Giemsa staining under a light microscope. Bars, 25 μm. One Caco-2 nucleus for each layer is indicated with the letter N. LPC-S01 adhesion was specific to Caco-2 cells: no adhesion was detected on the cover glass underlying Caco-2 cells (G; top part of the panel on the left). **(B)** Effect of *L. paracasei* strains on Caco-2 cells stably transfected with an NF-κB/luciferase reporter vector, in the presence of 5 ng/ml of IL-1β. Data in the histograms are the means (± standard deviations) from two independent experiments conducted in triplicate. RLU, relative luminescence units. Asterisks indicate statistically significant differences according to two-tailed unpaired Student's *t*-test: ^***^P < 0.001; ^**^P < 0.01; ^*^P < 0.05.

Subsequently, the ability of bacterial strains to adhere to the Caco-2 epithelial cell layer was tested. Only LPC-S01 displayed a marked adhesive phenotype, corresponding to an adhesion index (i.e., bacterial cells per 100 Caco-2 cells) of more than 2000 (Figure 5A). Also strain DG displayed adhesion ability, whereas strain Shirota was unable to adhere on Caco-2 cells, as observed in previous studies (Botes et al., 2008; Guglielmetti et al., 2008).

Caco-2 cell layer was also used to explore the immunomodulatory properties of *L. paracasei* strains by testing the effect of the microorganisms on NF-κB activation in presence of the pro-inflammatory cytokine IL-1β. To this aim, we employed a reporter cell line obtained by transfecting Caco-2 cells with a luciferase reporter vector induced by active NF-κB (Guglielmetti et al., 2010a). For all three *L. paracasei* strains, both bacterial cells and cell-free neutralized broths were able to significantly decrease the NF-κB-dependent production of bioluminescence. Particularly, we found that the highest ability to reduce NF-κB activation was exerted by DG and LPC-S01 cells, and by broth from Shirota culture (Figure 5B).

We also tested the ability of the bacteria under investigation to produce inhibitor molecules against pathogens by disc diffusion assay carried out by using cell-free MRS broths cultures. With the only exception of *L. plantarum* WHE 92 against *L. monocytogenes*, none of the neutralized broths was able to prevent pathogens' growth, suggesting that, plausibly, the only inhibitory molecules produced by tested *L. paracasei* strains were organic acids (Data not shown).

Afterwards, the *in vitro* probiotic properties of LPC-S01 and reference strains were assessed by testing their ability to produce acid from 44 different carbohydrate sources. The fermentation patterns of *L. paracasei* strains were very similar. In detail, the three tested strains fermented cellobiose, FOS, fructose, galactose, glucose, inulin, maltulose, mannitol, mannose, ribose, salicine, sorbose, sucrose, trehalose, and turanose (Supplementary Table 1).

In conclusion, the *in vitro* experiments carried out in this study showed that strain *L. paracasei* LPC-S01 possesses potential probiotic capabilities to be oral administered comparable to those of the probiotic commercial strains *L. paracasei* DG and Shirota. Notably, the survival rate of LPC-S01 to simulated gastrointestinal transit at pH 2 was significantly higher than that of the reference probiotic strain Shirota; in addition, strain LPC-S01 displayed marked adhesion ability, corresponding to an adhesion index higher than 2000.

We also studied the antibiotic resistance of *L. paracasei* LPC-S01by the microdilution assay recommended by International Organization for Standardization (ISO, 2010) with reference to the EFSA breakpoints for *L. casei*/*paracasei* (EFSA, 2012). The MICs for strain LPC-S01 was below the breakpoints for all tested antibiotics (Table 1). Finally, the safety of *L. paracasei* strains was also estimated by assessing the bacterial decarboxylation of amino acids, which generates biogenic amines in food (Deepika Priyadarshani and Rakshit, 2014). According to our experiments, none of the tested *L. paracasei* strains produced histamine, tyramine, putrescine and cadaverine (Data not shown), which are among the most common biogenic amines found in food products (Naila et al., 2010).

**Table 1:** **Antibiotic sensitivity of ***Lactobacillus paracasei*** LPC-S01 determined according to the microdilution assay recommended by EFSA (2012)**.

	**EFSA breakpoint**	**LPC-S01**
Ampicillin	2	2
Vancomycin	not required	>256
Gentamicin	32	32
Kanamycin	64	32
Streptomycin	Not required	32
Erythromycin	1	0.5
Clindamycin	1	0.062
Tetracycline	4	2
Chloramphenicol	4	4
Amoxicillin	Not indicated	1

*Data are reported as the lower concentration (mg/l) inhibiting bacterial growth (minimal inhibitory concentration, MIC)*.

### *L. paracasei* LPC-S01 persists in the gut of healthy adults

**Figure 6:**
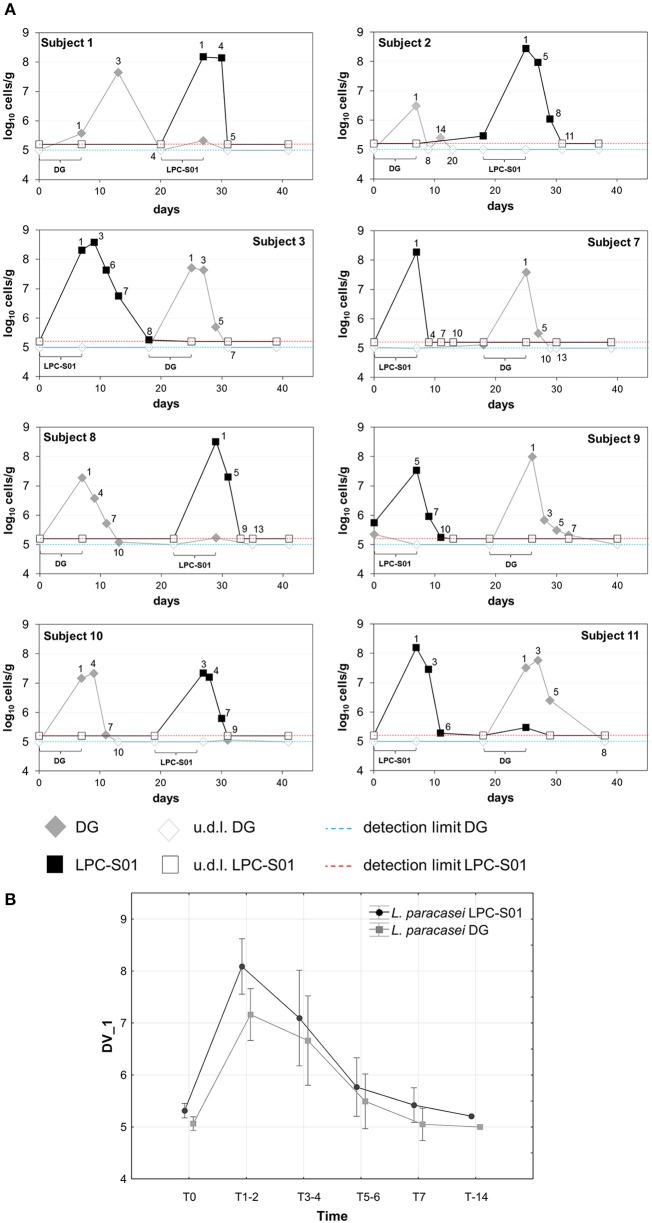
**Persistence of ***Lactobacillus paracasei*** in the gastrointestinal tract of healthy adults. (A)** Bacterial concentration of *L. paracasei* DG and LPC-S01 in the fecal samples collected during the study per single participant. **(B)** Average change in the fecal concentration of *L. paracasei* cells after the probiotic-intake period. Vertical bars denote 0.95 confidence intervals. Current effect according to repeated measures ANOVA performed to determine the statistical significance of the treatment × time interaction: *F*_(5, 65)_ = 0.794; *p* = 0.558.

The ability of *L. paracasei* LPC-S01 to colonize the human gastrointestinal tract was tested with a pilot intervention trial based on a double-blind, randomized, cross-over design, in comparison with the probiotic strain *L. paracasei* DG. We enrolled 11 healthy adult volunteers, who were asked to consume for 1 week a capsule per day containing LPC-S01 or DG cells. Out of 11 enrolled volunteers, 8 concluded the study. Three volunteers dropped out of the study due to non-conformity (*n* = 1) and protocol violation (missed probiotic consumption; *n* = 2) (Supplementary Figure 1). Capsules, which contained about 24 billion CFU of *L. paracasei*, were well tolerated by all participants and no adverse events were reported. In order to quantify *L. paracasei* cells in the fecal samples all over the trial, we used qPCR with primers targeting gene *welF* (specific for strain DG; Ferrario et al., 2014) and a gene coding for a hypothetical protein (specific for strain LPC-S01; primers designed in this study). According to repeated measure ANOVA, we did not find significant difference in the persistence of the two *L. paracasei* strains (Figure 6). Specifically, the concentrations of LPC-S01 and DG cells were above the qPCR detection limits for an average of 5 days from the end of treatment; in addition, *L. paracasei* cells were not detectable in the fecal samples after 7 ± 2 evacuations from the last capsule consumption (Figure 6A).

## Discussion

Bacterial vaginosis (BV) is one of the most common urogenital diseases affecting women of reproductive age (Allsworth and Peipert, 2007). BV is typically associated to the disruption of the healthy vaginal microbiota, which is normally dominated by lactobacilli (Ma et al., 2012). Due to the dominant role of dysbiosis in the onset of BV, probiotics, which are regularly used to prevent and treat dysbiosis in the intestine, have been proposed as a strategy to cure this urogenital condition and reduce its recurrence (Bodean et al., 2013). Several studies documented the beneficial effects on BV of viable *Lactobacillus* cells directly introduced in the vagina as suppository (Patel et al., 2008; Donders et al., 2010). Nonetheless, also the oral administration of probiotics has been demonstrated to be an efficient strategy to alleviate BV symptoms and prevent recurrence (Reid, 2006; Bodean et al., 2013). In fact, it was proposed that ingested lactobacilli, similarly to colonic pathogenic bacteria, once excreted from rectum, may ascend to the vagina through perineum (Reid et al., 2001; Bastani et al., 2012). Then, once in the vagina, lactobacilli create a protecting barrier against the translocation from the gut of possible detrimental bacteria such as *Prevotella* spp. or *E. coli*, and, at the same time, limit the possible migration of uropathogens from vagina to the bladder (Reid et al., 2001).

The oral administration of probiotics is a more practical route than their direct introduction in the vagina, and can also provide consumers with the gastrointestinal health benefits typically associated with the consumption *per os*. In this context, this study aimed to the identification of a probiotic strain possessing a wide range of properties, which may support its (i) industrial production, (ii) gastrointestinal administration, and (iii) vaginal colonization. To this aim, we focused our attention on *Lactobacillus paracasei*, a species characterized by marked ecological flexibility and extensive genetic diversity, which is widely used in industry (Cai et al., 2007). Particularly, we studied strain *L. paracasei* LPC-S01, which was originally isolated from the vagina of a healthy adult and has had a several-years history of commercial use as a vaginal probiotic for topical application in Europe. Specifically, the experiments carried out in this study were designed to evaluate if *L. paracasei* LPC-S01 has probiotic properties that can support its administration *per os*.

At first, the draft genome of LPC-S01 was analyzed by comparative genomics. We found the presence of numerous accessory genes (i.e., unique to this particular strain) that suggests the niche-generalists nature of LPC-S01; such potential ability to adapt to a wide range of environmental conditions is evidenced, for instance, by the presence of large chromosomal regions including genes for the utilization of numerous sugars (Figure 2C). Similar regions are also present in the genome of the probiotic strains DG and Shirota, which are of human intestinal origin. On the contrary, it was proposed that the adaptation of *L. paracasei* to the nutrient-rich milk environment had been gone along with extensive decay of genes involved in carbohydrate utilization (Broadbent et al., 2012). Accordingly, we found the lack of genes for sugar transport and metabolism in the chromosome of the dairy strains *L. paracasei* FMBr3 and N1115 compared to LPC-S01, DG, and Shirota strains (Figure 1).

The *in vitro* characterization of *L. paracasei* LPC-S01 was conducted in accordance with a procedure conventionally used to predict the probiotic potential of a microbial strain; such process consists in the assessment of sensitivity to acidity and bile, adhesion ability, pathogen inhibition, sugar fermentation profile, and immunomodulatory properties. In order to better predict the probiotic capabilities of LPC-S01, the reference strains Shirota and DG were also included in the study. *L. paracasei* Shirota is one of the most intensively studied probiotics (van den Nieuwboer et al., 2015; Wang et al., 2015) that has been used in the production of the fermented milk Yakult since 1935. *L. paracasei* DG is a bacterial strain with demonstrated probiotic properties (D'Incà et al., 2011; Tursi et al., 2013; Ferrario et al., 2014), which is included in Enterolactis®, one the most popular probiotic supplements in Italy. We observed that the *in vitro* performances of LPC-S01 were basically very similar to those of the reference strains. One exception was the significantly higher ability of LPC-S01 to survive in simulated gastric juice at pH 2 compared to *L. paracasei* Shirota, which is an acid-tolerant bacterial strain with demonstrated survivability to human gastrointestinal transit (Tuohy et al., 2007; Wang et al., 2015). In addition, LPC-S01 showed a much higher ability to adhere to Caco-2 epithelial cell layer than DG and Shirota. Bacterial adhesion properties mainly depend on the molecules exposed at the external cell surface (Guglielmetti et al., 2008; Polak-Berecka et al., 2014). Genome analysis revealed the presence in LPC-S01 of putative exopolysaccharide coding region (EPS2) containing a cluster of five genes which are not present in the other known *L. paracasei* genomes. Therefore, EPS2 could potentially support the synthesis of a peculiar exopolysaccharide molecule, which could plausibly contribute to the adhesion ability of the LPC-S01. Exopolysaccharides have been proposed to promote *Lactobacillus* adhesion (Ren et al., 2014), although, more frequently, the presence of a polysaccharide capsule has been shown to reduce adhesion by masking cell surface *Lactobacillus* adhesins determinants (Lebeer et al., 2012; Horn et al., 2013; Dertli et al., 2015), which are most commonly proteins (Lebeer et al., 2012; Polak-Berecka et al., 2014), (lipo)teichoic acids (Granato et al., 1999), and/or fatty acids (Polak-Berecka et al., 2014).

The acid tolerance and adhesion properties of LPC-S01 suggest that this bacterium might tolerate and adapt to the gastrointestinal environment. To test this hypothesis, we performed an intervention trial, in which we compared the gut persistence of *L. paracasei* LPC-S01 with that of strain DG. Following a daily consumption of 24 billion viable cells for 1 week, we estimated that strain LPC-S01 reached a concentration of about 10^8^ cells per g of feces, persisting above the detection limit (10^5^ cells per g of feces) for about 5 days, which is not significantly dissimilar to the persistence of the reference *L. paracasei* DG, a strain demonstrated to colonize the human gut of healthy adults and induce specific modifications in the microbiota composition (De Vecchi et al., 2008; Ferrario et al., 2014). Our data on *L. paracasei* persistence are in agreement with other studies, which observed that the persistence of *L. paracasei* Shirota in the gastrointestinal tract of healthy adults is lower than 1 week after cessation of the probiotic ingestion (Tuohy et al., 2007; Wang et al., 2015). The persistence of a certain probiotic strain in the gut plausibly depends on the evacuation frequency. In our study, we found persistence of strains LPC-S01 and DG as average for 7 evacuations. No data correlating the day of persistence with the number of evacuations were found in literature.

## Conclusions

The results of this study can be summarized as follows:

- according to comparative genomic analysis, *L. paracasei* LPC-S01 possesses the genetic features of a niche-generalist member of its species;
- *in vitro* tests evidenced that the probiotic properties of strain LPC-S01, with respect to those of the well-known probiotic references L. paracasei Shirota and DG, are equal for the bile tolerance and the reduction of NF-κB activation in Caco-2 cells, or superior for the tolerance to gastric juice and the adhesion to Caco-2 epithelial cells (Shirota is unable to adhere on Caco-2-cells);
- *L. paracasei* LPC-S01 is a safe bacterial strain for human consumption, which does not contain any acquired antibiotic resistance, does not produce biogenic amines and can be administered in high number (24 billion CFU) to healthy people without adverse events;
- when administered as capsule containing 24 billion CFU, *L. paracasei* LPC-S01 transiently colonizes the gastrointestinal tract of the host, persisting for at least 5 days after the end of a 7-days oral consumption (corresponding to about 7 evacuations) and therefore displaying colonizing performances not dissimilar to those of the probiotic commercial strain *L. paracasei* DG.

In conclusion, this study demonstrates that the vaginal isolate *L. paracasei* LPC-S01 possesses safety and functional properties that may support its use as probiotic to be administered *per os* for potential intestinal as well as vaginal applications. In this perspective, it would be of interest to carry out a clinical trial consisting of the oral administration to healthy adult women of *L. paracasei* LPC-S01 cells, in order to verify the potential ability of this probiotic bacterium to (i) modulate the intestinal and vaginal microbiota, and (ii) colonize the human vagina via gastrointestinal route.

### Conflict of interest statement

Research was financially supported by Sofar S.p.A., which is the company that produces and sells the probiotics products employed in the intervention study. WF is an employee of Sofar S.p.A. The other authors declare that the research was conducted in the absence of any commercial or financial relationships that could be construed as a potential conflict of interest.
