# Supplementary material for: The vaginal isolate Lactobacillus paracasei LPC-S01 (DSM 26760) is suitable for oral administration
Source: Front Microbiol. 2015 Sep 15;6:952. doi: 10.3389/fmicb.2015.00952 (PMC4569730; doi:10.3389/fmicb.2015.00952)

Supplementary Material

Supplementary Table 1. Phenotypic characterization of *Lactobacillus paracasei* strains based on acid production from 44 carbon sources. +, positive; –, negative; +/ –, weak activity.

	**LPC-S01**	**DG**	**Shirota**
D-Glucose	+	+	+
Lactose	+	-	+
D-Fructose	+	+	+
D(+)-Xylose	-	-	-
D-Galactose	+	+	+
Sucrose	+	+	+
L(+)-Arabinose	+/-	-	-
D(-)-Arabinose	-	-	-
D(+)-Trehalose	+	+	+
D(+)-Mannose	+	+	+
L-Rhamnose	-	-	-
Cellobiose	+	+	+
L(+)-Sorbose	+	+	+
D-Sorbitol	-	+	+
Inulin	+	+	+
Lactulose	+	-	+
D-Mannitol	+	+	+
FOS (Actilight)	+	+	+
Salicine	+	+	+
α-ciclodextrin	-	-	-
β-ciclodextrin	-	-	-
Arabinogalactan	-	-	-
Maltulose	+	+	+
D(+)-Turanose	+	+	+
Isomaltulose (palatinose hydrate)	-	-	-
GOS	+	+/-	+
D(-)-Ribose	+	+	+
Xylan from beechwood	-	-	-
Red Arabinan from sugar-beet	-	-	-
Arbutin	-	-	-
D(-)-Tagatose	+	+	-
D(+)-Fucose	-	-	-
D(+)-Raffinose	-	-	-
Glycerol	-	-	-
D-Gluconic acid	+/-	+/-	-
Meso-Erythritol	-	-	-
Melibiose	-	-	-
Myo-Inositol	-	-	-
Inositol	-	-	-
D-Glucoron-γ-Lactone	-	-	-
Glycogen	-	-	-
Esculin	+/-	-	-

Supplementary Figure 1. Schematic of study design and flow. V0–V5, visits before the run-in period, before the first treatment, after the first treatment, before the second treatment, after the second treatment, and after final follow-up, respectively.

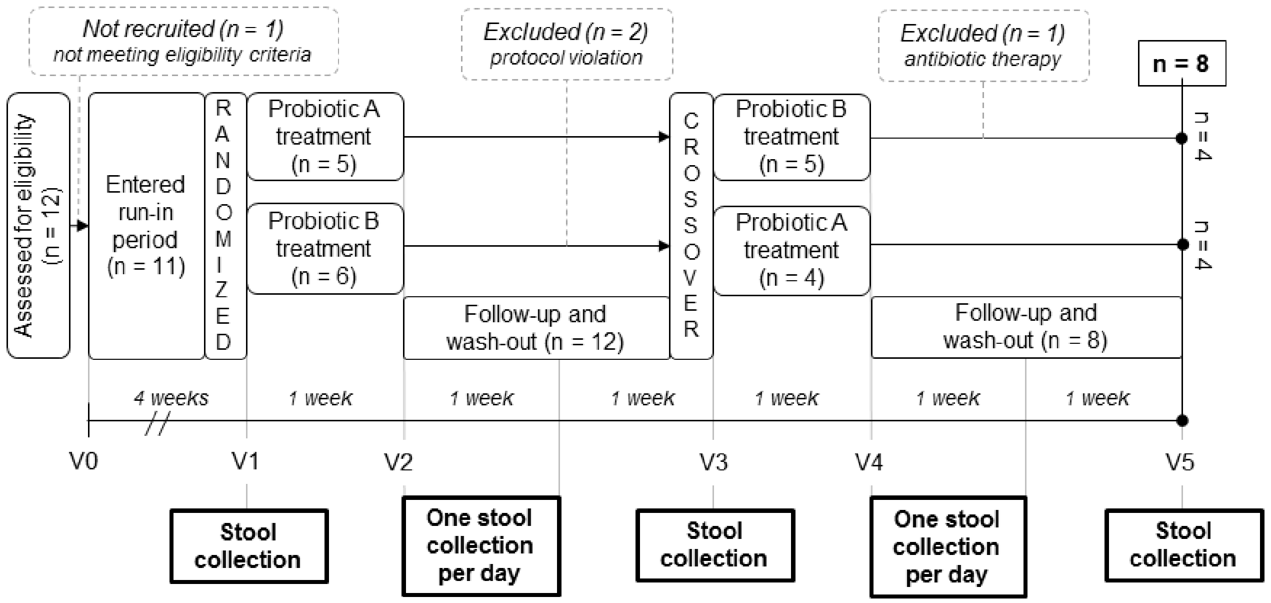

Supplementary Figure 2. Tolerance of *Lactobacillus paracasei* strains to simulated gastric juice at pH 3 (90 min incubation) and bile (180 min incubation). Dashed lines refer to controls (i.e., bacterial cells incubated in phosphate buffer, pH 6.5). Data are reported as the number of viable bacterial cells (CFU) plotted on a semi-logarithmic diagram. Vertical bars at each point refer to standard deviation calculated on three independent experiments conducted in duplicate.

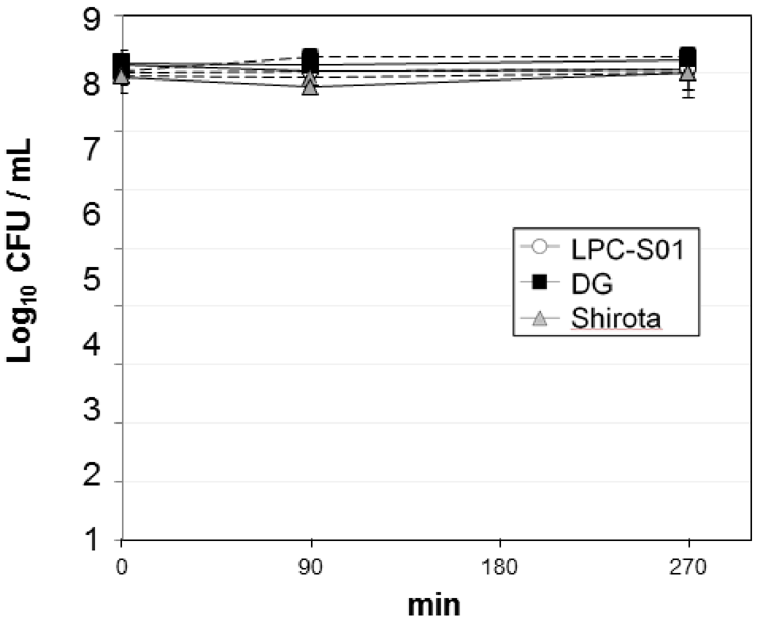

Supplementary Figure 3. Growth of *Lactobacillus paracasei* strains in MRS broth supplemented with increasing concentrations of bile (Oxgall).

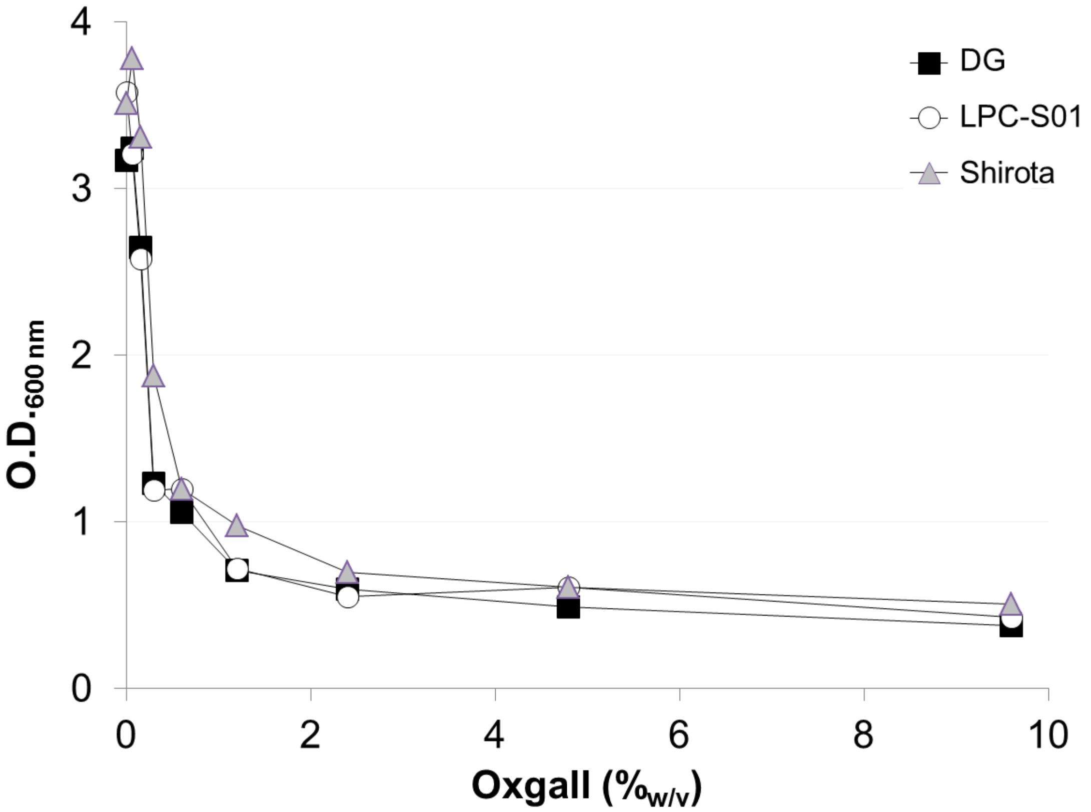

Supplement: Supplementary file 1 [file DataSheet1.PDF]
